# The Bølling–Allerød Transition in the Eastern Baltic: Environmental Responses to Climate Change

**DOI:** 10.3390/biology12060821

**Published:** 2023-06-05

**Authors:** Olga Druzhinina, Anna Rudinskaya, Ksenia Filippova, Lyudmila Lazukova, Nadezhda Lavrova, Anton Zharov, Ivan Skhodnov, Aleksey Burko, Kasper van den Berghe

**Affiliations:** 1 Faculty of Geography, Herzen State Pedagogical University of Russia, 191186 Saint-Petersburg, Russia; anna.rudinskaya@igras.ru (A.R.); xenia.filippova@igras.ru (K.F.); lazukova@yandex.ru (L.L.); alesburk@mail.ru (A.B.); 2 Institute of Geology, Karelian Research Centre, Russian Academy of Sciences, 185910 Petrozavodsk, Russia; lavrova@krc.karelia.ru; 3 A.N. Severtsov Institute of Ecology and Evolution, Russian Academy of Sciences, 119071 Moscow, Russia; antzhar.ipee@yandex.ru; 4 Baltic Archaeology Research Centre, 236000 Kaliningrad, Russia; ivanskhodnov@gmail.com; 5 FindX Research Center, 8031 VK Zwolle, The Netherlands; berghekj@hotmail.com

**Keywords:** paleoclimate, Bølling, Older Dryas, Allerød, lithology, diatoms, pollen, Lateglacial, Baltic Ice Lake, south-eastern Baltic

## Abstract

**Simple Summary:**

During the last glaciation, the nature of the northern hemisphere of the Earth underwent significant changes. Nonetheless, it underwent no less serious changes after the melting of the glacier, when, under the conditions of climate mitigation, the gradual formation of modern landscapes began in the spaces freed from ice. This study is devoted to the reconstruction of the post-glacial environment in one of the regions of Eastern Europe, in the south-eastern part of the Baltics. The uniquely preserved deposits of one of the post-glacial basins discovered here made it possible to reconstruct in detail the changes in climate, vegetation, and aquatic organisms in the time interval of 14–13.4 thousand years ago. The study revealed that during this period there were short-lasting climate fluctuations (warmings and coolings), which caused repeated changes in all components of the local nature. The results of the study contribute to understanding the complex processes of planetary climate formation, as well as the impact of climate on nature both at local and global levels, which is necessary not only for understanding the past, but also for predicting the future of all living organisms on the planet.

**Abstract:**

This paper presents the results of a study on the Kulikovo section (south-eastern Baltic Sea coast), a sediment sequence exposing deposits of a post-glacial basin that existed along the edge of the glacier in the Late Pleistocene. The research was targeted at the reconstruction of the dynamics of the local environmental systems in response to climatic oscillations of the Lateglacial (the Older Dryas—first half of the Allerød). The evolution of the local biotic components on the territories of the Baltic region after the ice retreat is still poorly understood. Data from geochronological, lithological, diatom, algo-zoological and palynological analyses provide a reconstruction of local aquatic and terrestrial biocenoses and their response to short-term warmings and coolings that took place 14,000–13,400 cal yr BP. This study has demonstrated that, during the Older Dryas and first part of the Allerød (GI-1d and GI-1c), the aquatic and terrestrial environment of the Kulikovo basin underwent several changes, resulting in eight stages of the basin evolution, most probably related to the short-term climatic fluctuations that could have had a duration of several decades. The data obtained in this study have revealed the fairly dynamic and complex evolution of the pioneer landscapes, as indicated by the changes in the hydrological regime of the area and by the traced successions of plant communities from the pioneer swampy vegetation to park and real forests towards the middle of the Allerød.

## 1. Introduction

The Bølling–Allerød warming event of the last deglaciation was the first northern hemisphere abrupt warming that occurred between Heinrich Stadial 1/Oldest Dryas and the Younger Dryas, 14,700–12,850 cal yr BP [1,2]. Previously being described as consisting of three phases or chronozones (the Bølling and Allerød warm interstadials and the Older Dryas cold stadial in between), later it was subdivided into five to seven subevents according to high-resolution records from continental Europe and Greenland [1]. Detecting these centennial-scale events is very challenging, first of all due to relatively low temporal resolution of the majority of terrestrial records and because some proxies are not sensitive enough to respond to these abrupt cold spells. At the same time, a detailed study of short-term climatic Bølling–Allerød oscillations is a necessary task, without solving which it is impossible to approach the understanding of the complex dynamics of the Lateglacial environmental systems.

**Figure 1:**
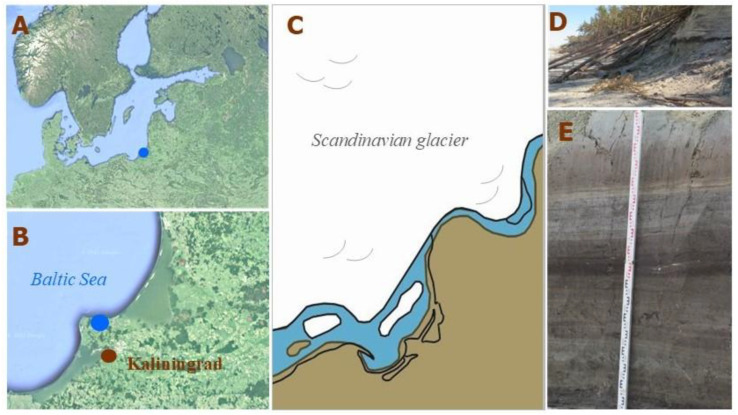
(**A**) Location of the studied site in Europe; (**B**) location of the studied site in Kaliningrad region (south-eastern Baltic, Russia); (**C**) reconstruction of the paleogeographic situation in the south-eastern Baltic about 14,500 cal yr BP [8]; (**D**) erosional coastal ledge at Kulikovo before sampling; (**E**) Kulikovo section during sampling.

One of the rare paleoenvironmental records where a high-resolution research is possible was discovered in 2022 at the south-eastern coast of the Baltic Sea in Kulikovo (Kaliningrad region of Russia). The section, nearly 2 m thick, embraces the time period between 14,000 and 12,000 cal yr BP and allows a zoom-in into the period, when the formation of the pioneer landscapes started on the spaces freed from ice (Figure 1). 

The south-eastern part of the Baltic region belongs to the areas covered by glaciers during the Weichselian (Valdai) glaciation maximum. Since the beginning of deglaciation in the Late Pleistocene, the natural environment of this region has undergone significant changes [3,4,5,6,7,8].

The general outline of the paleogeographical background of the south-eastern Baltic for the study period is given in [8,9]. The beginning of the present-day Baltic Sea is associated with the recession of the last Scandinavian continental glacier, which began about 20,000–19,000 cal yr BP, and according to [9] the south-eastern Baltic territory was free of ice about 16,000–14,000 cal yr BP. Waters of the melting and retreating glacier gathered in front of it, creating ice-dammed lakes. Probably, local marginal lakes located at that time in the Bornholm and Gdańsk basins merged at about 14,500–14,000 cal yr BP and formed the Baltic Ice Lake (BIL) [8]. The Kulikovo section represents one of the separate smaller basins gathering water from the melting dead ice and newly forming a hydrographical net, and existing on the land along the coast of the BIL. The size and water level fluctuations of the latter strongly depended on the oscillations of the glacier front in Scandinavia, opening and closing the connection with the ocean, which resulted in a complex dynamic of the whole system. Post-glacial isostatic rebound was adding more complexity to the evolution of the coastal landscapes, as according to [8] the Earth’s crust after deglaciation within the area of the southern Baltic Sea was 90 m lower than at present and uplifted in a short time. 

Apparently, the Kulikovo section reveals the sediments of one of the shallow post-glacial basins formed on land as a result of ice degradation. The uniqueness of the section lies in the fact that most of the post-glacial reservoirs that existed along the Baltic coast were later destroyed during transgressions and regressions of the Baltic Sea. According to the reconstructions of the ancient coastlines of the BIL and the Baltic Sea, after the retreat of the glacier (14,500–14,000 cal yr BP), as well as in the early Holocene (the Yoldian and Ancylus stages of the Baltics), a now flooded strip several tens of kilometers wide belonged to the land [7,9]. It is possible that in the period between 14,000 and 11,600 cal yr BP there were transgressions of the BIL, during which an exchange of water between the latter and inland water bodies was possible. The Holocene erosion of the coast and neotectonic processes have led to the fact that the remains of sediments of the Kulikovo basin are currently exposed in the erosional coastal ledge of the Baltic Sea. The reconstruction of the size and other parameters of the former basin is subject of a separate study. 

The Kulikovo section is 192 cm thick. The deposits were studied by the methods of lithological (grain size, loss-on-ignition, carbonate content, and magnetic susceptibility), diatom, palynological, and algo-zoological microfossil and geochronological analyses. The aim of the study was to reconstruct the paleoenvironment of the basin and the surrounding landscapes and to reveal the reaction of the local environmental systems to the major climatic changes of the Lateglacial.

At this stage of research, the most detailed data were obtained for the lower part of the section (192–141 cm), comprising the Older Dryas and the first half of the Allerød. The current paper is devoted to this interval of the section and presents the first results of the comprehensive study.

## 2. Materials and Methods

### 2.1. Fieldwork and Sampling

The sampling location is situated at the erosional ledge of the Sambian Peninsula of the south-eastern Baltic Sea coast (54.932° N, 20.357° E). The sediments were placed into so-called “pollencolumn boxes”, metal boxes with a 7 cm diameter and 50 cm long, and transported to laboratories for further processing and analyses. Sampling was carried out depending on visible layering of the sediment (each 1–3 cm).

### 2.2. Radiocarbon Dating

Five samples of the sediments from the entire section (0–192 cm) were subjected to radiocarbon dating using the automated graphitization system Ionplus AGE-3 and SSAMS (Single Stage Accelerator Mass Spectrometer) in the Lund University Radiocarbon Laboratory. The gyttja sediment (one sample) and wooden residues (four samples) were dated (Table 1). The calculation of the ^14^C age is based on the half-life of 5568 years. In the uncertainty statement (±1 SD), statistically accessible contributions from the measurements of samples, standards and background were included. The age determination is based on 95% of the activity of the NBS oxalic acid standard. All ^14^C ages were ^13^C corrected for the deviation from the agreed standard value of the ^13^C/^12^C ratio. The age–depth model (ADM) was built using the rbacon program 3.1.0. [10]. All dates were calibrated to calendar years before present (BP) using the IntCal20 calibration curve [11].

### 2.3. Lithological Analysis

Twenty-four samples from the lower part of the section (141–192 cm) were studied. Analytical characteristics included the particle size distribution of sediments, the content of organic matter and CaCO_3_, and the magnetic susceptibility values.

The grain size analysis was performed on a Malvern Mastersizer 3000 laser diffractometer with a Hydro EV receiver after preliminary sample preparation, including the removal of the carbonate component of the sediment with a 10% solution of hydrochloric acid HCl (reaction in test tubes for 1 h) and removal of organic matter using hydrogen peroxide H_2_O_2_ (reaction for at least 6 h in a water bath at a temperature of 90 °C). After that, the samples were continuously rotated for 12 h with a sodium pyrophosphate Na_4_P_2_O_7_ solution. Then the material was dispersed using an ultrasonic bath (360 W) for 30 min before the measurement on a laser diffractometer. The particle size distribution was determined using the Mie diffraction model [12]. The lithological characteristic of the mineral content of the sediment was determined using GRADISTATv9.1 (http://www.kpal.co.uk/gradistat.html (accessed on 5 March 2023)) and the particle size distribution classification Microsoft Excel 2007/2010 program (http://www.kpal.co.uk/particle_size.html (accessed on 5 March 2023)). This is a revised system of size class nomenclature [13,14]. The scheme has five first-order size classes (boulder, gravel, sand, silt, and clay), each of which has five second-order subdivisions with limits defined at one phi intervals, and is suitable for use in environmental and engineering investigations. 

Loss on ignition (LOI). The method is based on sequential heating of the samples in a muffle furnace at three temperatures (105 °C (dry weight), 550 °C, and 950 °C) and weighing on an electronic scale after each heating step [15]. The content of CaCO_3_ is determined as LOI 950×2.27.

Mass magnetic susceptibility (MS) was measured using a ZHinstruments SM 150 L susceptibility meter operating at a magnetic field strength of 320 A/m and two frequency modes (500 and 4000 Hz). No frequency dependence was revealed. Sample preparation included preliminary drying of the material at a temperature of 40 °C.

### 2.4. Diatom Analysis 

Diatom analysis was performed on 24 samples from the interval 142–191 cm. Slides were prepared according to the standard procedure [16]. One lycopodium tablet was added to each sample (Batch 280521 291–13,761 spores in one tablet without taking into account the standard deviation) to calculate the weight concentration of valves (valves/g of the dry material). In each slide, 100 to 1600 valves were identified. Differences in the number of identified valves for each preparation are associated with different saturations of the samples with valves. Diatom species were identified using taxonomic works [17,18,19,20,21,22]. The names of the identified diatom species were verified using the resource Algaebase.org [17]. 

In order to describe the paleoecological conditions of the water basin, diatom species were classified into ecological groups’ salinity [23]. According to water salinity, the following groups of diatoms were distinguished: brackish (water salinity 5 to 20‰) and freshwater diatoms. The freshwater diatom group was subdivided into halophilous diatoms, which spread in very low salinity environments; indifferent, whose reproduction is best in freshwater conditions, although they are able to live in water with high mineralization; and halophobic species, which prefer exclusively freshwater habitat conditions. Depending on the trophy of the reservoir, the diatom species were subdivided into oligotrophic, which spread in water bodies with cold, well-oxygenated clear water and a low content of dissolved nutrients; eutrophic, which spread in water bodies characterized by high productivity and a high content of biogenic elements; mesotrophic; and a group of diatoms indifferent to trophy of the habitat. 

The diatom diagram was compiled using the TILIA 2.6.1 software package [24]. It depicts taxa, the share of which exceeds more than 2% of all species.

### 2.5. Algo-Zoological Microfossil Analysis 

Nine samples were studied by algal and zoological analysis. Native ground was stirred in a small volume of filtered water, and water–glycerol-based temporary slides were made for microscopic study. The remains of algae and aquatic invertebrates were counted using each subfossil remains as an arbitrary unit of a corresponding group of organisms. Following N.N. Smirnov’s method [25], analysis was divided into three stages (“levels”). The remains were split up into various taxonomic groups and analyzed. At level I, the remains of algae of each large taxon, e.g., Bacillariophyta, Desmidiales, and Chlorophyta, were counted, while the remains of invertebrates were counted totally (Animalia). At level II, only zoogenic remains of Cladocera, Ostracoda, Chironomidae, and Spongia were analyzed, splitting them up into groups of a high taxonomic rank. At level III, the remains of the indicator group, Cladocera, were identified to a species or a group of species, if they could not be identified more accurately. At each analytical level, at least 100 (over 200, if possible) subfossil microremains were counted [26]. The preparations were analyzed under transmitted light of Olympus CX and Biomed-3 light microscopes, using magnifications of 10 × 20 and 10 × 10. In some cases (when the concentration of remains in a ground sample was low), the remains of invertebrates, particularly Cladocera, were analyzed by washing ground samples in running water on a 100 µm mesh mill gas sieve before making slides for microscopy. At level I, analysis was always performed by examining unwashed material to avoid the loss of the finest remains and the distortion of their native proportions. 

### 2.6. Palynological Analysis 

Twenty-four samples from the lower part of the section were studied. The sub-samples of 3 cm^3^ were prepared using a standard chemical procedure [27], including treatment of the sediments with a heavy liquid (CdI_2_ + KI). Lycopodium spores were added in order to calculate pollen concentrations. Pollen identification was based on [28]. In most of the samples, the number of counted terrestrial pollen grains exceeded 400, with the exception of several samples where the number of pollen grains was lower. 

For calculation and presentation of the pollen data, the programs TILIA and TILIA-graph were applied [24]. Along with the visual inspection, a stratigraphically constrained cluster analysis (CONISS) was used for the subdivision of pollen zones [24].

## 3. Results

### 3.1. Radiocarbon Dating

**Figure 2:**
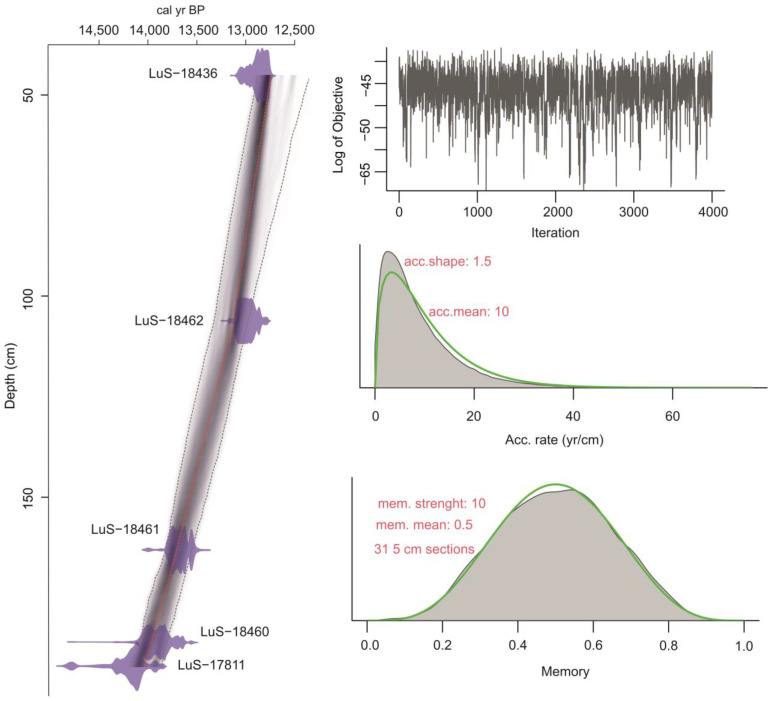
Age–depth model for the study section.

The geochronological results of the entire sediment section (192–0 cm) are presented in Figure 2 and Table 1.

The model’s output contains ages calculated for each centimeter of the section. The applied sampling interval of 1–3 cm roughly corresponds to 6–30 years. The model graph shows no outliers—all the dates fall into the 95% CI range. According to ADM, the sedimentation starts at 14,038 ± 140 cal yr BP. The uncertainty for the lowest part of the section (192–145 cm) is ±130–160 years. It rises upwards to ±170–190 years at the depths of 145–90 cm and to ±200–240 years at the depths of 90–45 cm. ADM was not built for 45–0 cm due to an expected significant error.

### 3.2. Lithological Analysis

**Figure 3:**
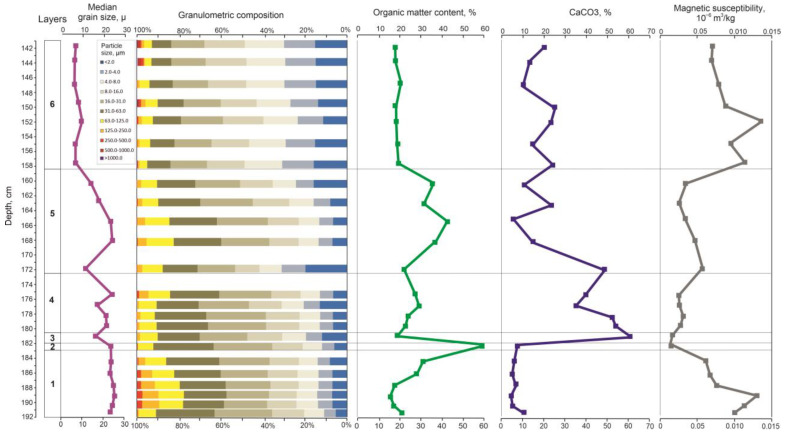
Results of the lithological analysis of the Kulikovo section.

As a result of comprehensive lithological analysis, the following layers were identified (Figure 3).

Layer 1, 192–183 cm. Slightly clayey, sandy, very coarse silt. The silt fraction prevails (63.6–80.3%), the content of sand is 13.9–22.3% and 8.7% at 191–192 cm, and clay is 11.0–14.1%. The content of organic matter increases from the bottom of the layer to the top from 10.7–12.6% to 25.1%, and the CaCO_3_ content varies from 7.8 to 13.7%. MS decreases from 0.13 to 0.07 × 10^−6^ m^3^/kg. Average median grain size is 23.9 µm.

Layer 2, 183–182 cm. Slightly sandy, slightly clayey, very coarse silt. The content of silt is 80.4%, sand—7.7%, and clay—11.9%. The content of organic matter reaches 54.6%, and the content of CaCO_3_ is about 10%. MS is 0.02 × 10^−6^ m^3^/kg. Median grain size is 23.3 µm.

Layer 3, 182–180.5 cm. Slightly sandy, slightly clayey, coarse silt. The content of sand is about 10%, silt is 70.6%, and the content of clay is 19.5%. The content of organic matter decreases to 13.6%, and the content of CaCO_3_ increases to 61.6%. MS is 0.03 × 10^−6^ m^3^/kg. Median grain size is 17.1 µm.

Layer 4, 180.5–172.5 cm. Slightly sandy, slightly clayey, coarse silt. The sand fraction is up to 8.6–15.7%, the silt varies from 70.2 to 78.6%, and the content of clay particles varies from 12.8 to 20.4%. The content of organic matter increases from 17.3 to 23.5%, and the content of CaCO_3_ decreases from 54.9 to 37.1%. MS is 0.03–0.04 × 10^−6^ m^3^/kg. Median grain size varies from 17.4 to 23.7 µm.

Layer 5, 172.5–158.5 cm. Slightly sandy slightly clayey, coarse silt. The silt fraction prevails (56.6–74.2%), sand—up to 17.2%, and clay fraction—13.7–31.2%. The content of organic matter increases from 15.9% to 30–36%, and CaCO_3_ changes from 49.5% in the bottom part of the layer through 8.1% in the middle part to 13–25% in the upper part of the layer. MS is 0.03–0.06 × 10^−6^ m^3^/kg. Median grain size varies from 13–15 to 20–23 µm.

Layer 6, 158.5–141 cm. Slightly sandy, clayey, fine and medium silt. The content of the silt fraction varies from 63.1 to 69.1%, the content of sand varies from 4.8 to 7.4%, and the clay content—from 23.5 to 30.9%. The content of organic matter varies from 12.2 to 15.4%, and the content of CaCO_3_ varies from 16.0 to 27.5%. MS increases from 0.14 to 0.08 × 10^−6^ m^3^/kg. Median grain size is about 8.3 µm, and at the level 153.0–149.5 cm increases to 10–11 µm.

### 3.3. Diatom Analysis

**Figure 4:**
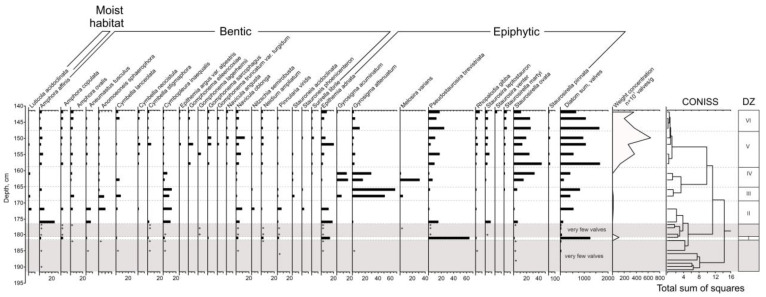
Results of the diatom analysis of the Kulikovo section.

The following diatom zones were identified (Figure 4).

192–182 cm: very few valves are found (from 1 to 30 for the sample).

DZ-I, 182–180.5 cm: the majority are epiphytic species, and, among them, the halophilous eutrophic species *Pseudostaurosira brevistriata* (64%) and the indifferent eutrophic thermophilious species *Epithemia adnata* (13.5%) dominate. Weight concentration is 1 million valves per gram of dry sediment.

180.5–176 cm: very few valves are found (from 20 to 50 for the sample).

DZ-II, 176–169 cm: the benthic species dominate; most of them are indifferent species spread in ware bodies of different trophicity: *Amphora affinis* (its content decreases from 23 to 9%), halophobic mesotrophic *Cymbopleura inaequalis* (10–13%) and the eutrophic indifferent species *Navicula oblonga* (5–9%). The majority of the epiphytic species is *Epithemia adnata* (from 11 to 18%). Weight concentration increases from 50 to 110 thousand valves per gram of dry deposits up the DZ-II.

DZ-III, 169–165 cm: epiphytic species predominate, represented mainly by valves of two eutrophic species—*Gyrosigma attenuatum* (from 50 to 66% of valves) and *Gyrosigma accuminatum* (7% of valves in the bottom part of diatom zone). *Cymbopleura inaequalis* (9–13%) dominates among the benthic species. Weight concentration decreases from 100 to 50 thousand valves per gram of dry deposits up the DZ-III.

DZ-IV, 165–158.5 cm: half or slightly more are epiphytic, valves of halophobic mezotrophic species *Staurosirella ovata* (from 15 to 32% of valves), halophilous eutrophic species *Melosira varians* (its content in the bottom part of the DZ-IV is 31%, then its content sharply decreases to 1%), and the valves of the species *Gyrosigma attenuatum* (from 16 to 29%) and *Gyrosigma accuminatum* (15–17%) dominate. The content of mesotrophic species increases up the DZ-IV, while the content of eutrophic species, on the contrary, decreases.

DZ-V, 158.5–148 cm: epiphytic diatoms predominate; most of them are represented by *Staurosirella ovata* (from 13 to 44% of valves), and *Pseudostaurosira brevistriata* (the content varies from 3 to 17%). Among benthic diatoms, valves of *Navicula oblonga* (from 4 to 13%) dominate. Brackish diatoms for the first time appear in the section (1–3%). Weight concentration increases from 1.3 to 6.4 million valves per gram of dry deposits up the DZ-V.

DZ-VI, 148–141 cm: Epiphytic diatoms predominate; most of them are represented by valves of the species *Pseudostaurosira brevistriata* (13–24%) and *Staurosirella ovata* (their content decreases up the zone from 26 to 11%). Brackish diatoms slightly increase (1–6%). The weight concentration varies from 2 to 3.7 million valves per gram of sediment.

### 3.4. Algo-Zoological Analysis

The analysis of the algo-zoological samples (AZS) revealed the following results:

AZS-1, 190–189 cm: an abundance of amorphous organic matter (plant detritus) and mineral particles. The interval yields no remains of algae and aquatic invertebrates.

AZS-2, 183–182 cm: plant detritus and the remains of microfossils of invertebrates are scarce. The chitin fragments of insects and, presumably, soil ticks are most common. Few Desmidia algae of *Cosmarium* sp., and the cocoons of Turbellaria and the shells of Testacea are present. Freshwater molluscs are abundant: *Pisidium* sp., Planorbidae, and Bithyniidae.

AZS-3, 182–181 cm: algal flora and exuberant aquatic fauna are present. Algal flora consist of diatoms (preserved mostly as armor fragments with signs of dissolution) and Desmidia of *Cosmarium* sp. and *Euastrum* sp. Zoogenic remains are dominated (mainly as fragments and less commonly as intact valves) by freshwater ostracod shells, Cladocera exoskeletons and Testacea shells. Cladocera display abundant littoral fauna, and the remains of pelagic taxa (*Bosmina coregoni* s.l. and *Daphnia* (D.) sp.) collectively make up no more than 7%. Presence of *Alonella nana*. An abundance of molluscs. 

AZS-4, 174–173 cm: algal flora consist of the desmidian algae Cosmarium sp., which make up over 30% of all subfossil remains. No remains of diatoms are revealed. Pediastrum sp occur as single algae. The spicules of the sponge Spongilla sp. are found for the first time. Ostracods are less abundant than in the previous layer. The percentages of Bosmina coregoni s.l. and Acroperus harpae among littoral-thicket species in the remains of Cladocera are observed to increase. 

AZS-5, 167–166 cm: Desmidia (*Cosmarium* sp.) are less abundant than those in the previous layer. *Pediastrum* sp. and the oogonia of charophytes occur as single plants. The composition and ratio of Cladocera species have not changed substantially in comparison with those in the underlying layer. Shells of *Pisidium* sp. present in large amounts.

AZS-6, 160–159 cm: the concentration of subfossil microremains has decreased markedly. Algal flora are dominated by diatoms (96% of all subfossil remains), and *Cosmarium* are less common than those in underlying layers. The percentage of ostracods has abruptly decreased. They are present solely as fine fragments, whereas the spicules of the sponge *Spongilla* sp. in this layer increase considerably and continue to do so in overlying layers. Molluscs are dominated by *Pisidium* sp. and Bithyniidae. 

AZS-7, 8, 9; 154–153, 149–148, 144–143 cm: diatoms represent over 90% of algal remains. *Cosmarium* sp. and *Pediastrum* sp. occur as single algae. The contribution of ostracods, occurring solely as fine fragments, decreases abruptly. The most abundant zoogenic microremains consist of the spicules of the sponge *Spongilla* sp. (they make up 56–68% and tend to become more abundant). The contribution and species composition of Cladocera against the succession of an invertebrate group dominating in taphocenosis have not changed greatly. The remains of fauna in the open lacustrine conditions make up about 20% of Cladocera remains, except for the top of the layer (144–143 cm), in which the percentage of *Bosmina coregoni* s.l. decreases, and the contribution of some thicket species increases. Molluscs disappear at the depth of 154 cm but present again at the second part of the interval starting from 149 cm.

### 3.5. Palynological Analysis

**Figure 5:**
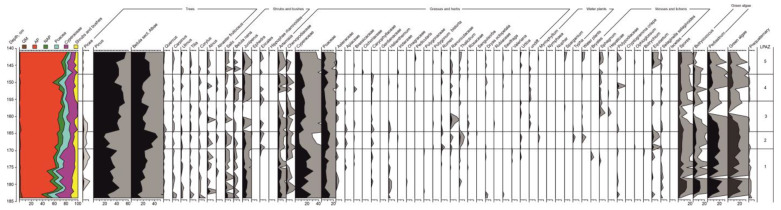
Results of the palynological analysis of the Kulikovo section.

The following palynological zones were identified (Figure 5).

LPAZ 1, 184–169 cm. Samples in the lowest part of the section (192–184 cm) contain insufficient amounts of pollen and spores; *Pinus*, *Betula*, *Artemisia*, Cyperaceae, and *Selaginella selaginoides* are identified.

The contribution of AP varies from 35% to 60% throughout the zone. There is a dominance of *Pinus* (up to 45%) and *Betula* sect. Albae (up to 26%). Continuous curves of *Picea* (3%), *Salix* (5%), and *Betula nana* (2%). *Juniperus* present (8%). At the top of the zone, *Ericales* appear. Among herbs, the highest numbers are of Cyperaceae (32%) and Poaceae (15%); there is also a significant presence of *Artemisia* (8%) and Chenopodiaceae (3%). *Helianthemum*, *Rumex*, *Thalictrum*, and *Dryas octopetala* present. There is a maximum amount of *Pediastrum* (25%) and green algae in general. Redeposited pre-Quaternary sporomorphs are also present. 

LPAZ 2, 169–165 cm. There is a sharp decline of *Pinus* (to 20%) and peak of *Betula* sect. Albae, which has the highest value through the studied sequence (46%). There are peaks of *Ericales*. There is a strong decrease of Cyperaceae (7%). Poaceae vary from 5% at the bottom to 10% at the top of the zone. There are remarkable peaks of *Thalictrum* (2%), *Typha* (0.5%), and *Equisetum* (4%).

LPAZ 3, 165–156 cm. There is a substantial increase of *Pinus* (to 55%) and a decline of *Betula* sect. *Albae* (to 20%), with two short-lasting peaks. *Picea* disappears in the second part of the zone. *Betula nana*, *Juniperus*, and *Ericales* have the lowest values in the upper part of the zone as well. Poaceae recover (up to 20%), while *Artemisia* and Chenopodiaceae decline towards the top of the zone. Ranunculaceae have a maximum value (3%), followed by a peak of *Sphagnum* (2%). Equisetum disappears throughout the zone and green algae have a minimum value too. There are single redeposited pre-Quaternary sporomorphs.

LPAZ 4, 156–148 cm. The contribution of AP is high (up to 75%) but passes a sharp and short-lasting decline (to 55%) in the upper part of the zone. *Pinus* decreases to 35% and then rises again up to 45%. Simultaneously, the value of *Betula* sect. Albae increases in the upper part of the zone (34%). *Betula nana* has a maximum value throughout the sequence (3%). *Juniperus* recovers again (up to 6%) as well as *Artemisia* (6%), Chenopodiaceae (3%), and Poaceae (13%). *Sphagnum* is constantly present (1%). Polypodiaceae have a peak (3.5%).

LPAZ 5, 148–141 cm. There is a high value of AP (80%). Both *Pinus* and *Betula* sect. Albae have a tendency to decrease towards the middle of the zone and then increase simultaneously (up to 56% and 25%, respectively). *Picea* re-appears (<1%). *Salix* (up to 3%), *Betula nana* (2%), *Juniperus* (4%), *Artemisia* (3%), and Chenopodiaceae (2%) have higher values in the lower part of the zone and decrease towards the top. In the upper part of the zone, values of water plants (1%) and *Sphagnum* (2%) increase, while Polypodiaceae (2%) and *Equisetum* (4%) have the second remarkable peak throughout the sequence.

## 4. Discussion

**Figure 6:**
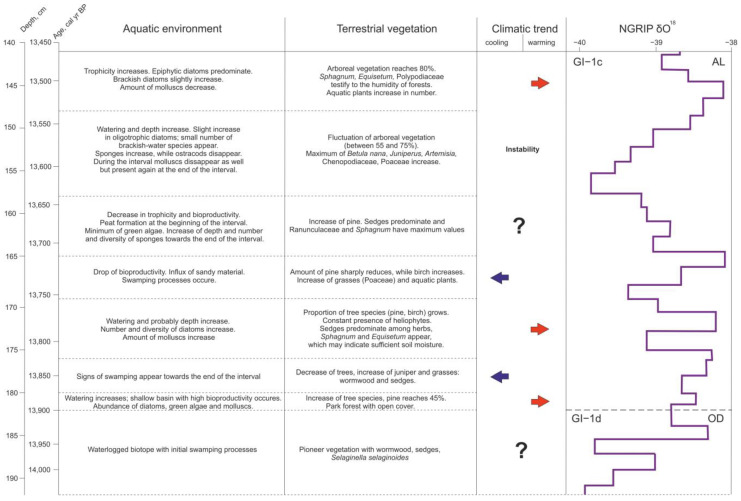
Stages of aquatic and terrestrial environment development during the Older Dryas—first half of the Allerød inferred from the Kulikovo section study.

The results obtained at this stage of the research make it possible to reconstruct the following stages in the development of the basin and adjacent landscapes during the Older Dryas—first half of the Allerød. According to ADM, the sedimentation at the study area started at 14,038 ± 140 cal yr BP (Figure 6).

At the earliest stage in the interval, 14,038–13,904 (±140), preceding the formation of the basin as such, the study area was probably a waterlogged biotope with initial swamping processes. The samples for pollen analysis did not contain sufficient amounts of pollen grains and this fact, along with the higher proportion of re-deposited microfossil material, does not allow reconstructing the vegetation cover with certainty; can, however, assume the presence of a pioneer vegetation with *Artemisia*, Cyperaceae, and *Selaginella selaginoides*. 

Then, approximately 13,904 ± 140 cal yr BP, the watering of the site increases and a shallow basin with high bioproductivity appears, characterized by an abundance of diatoms and green algae. The freshwater molluscs encountered (*Pisidium* sp., Planorbidae, and Bithyniidae), testify to standing or slowly flowing shallow waters, and they are typical for plant rich habitats. While Planorbidae have adapted to exist in a wide variety of conditions, Bithyniidae can withstand low temperatures, although the optimum water temperature for their existence is 15–22 °C [25]. Probably, local warming occurred during this period, since the spore-pollen data show some increase in the number of tree species, primarily pine (up to 45%). In the debatable issue of the presence or absence of tree species in the vegetation cover in this region of Europe in the Lateglacial, we rely on the regional studies considering the pollen accumulation rate and plant macrofossil data [29,30,31], and assume the possibility of the existence of the so-called park forests with open cover, at least during periods of climate amelioration, starting from 13,900 cal yr BP. This is also evidenced by the data of phytolith studies of the Kulikovo section (in prep.), which recorded the presence of phytoliths of coniferous trees in layers dating back to approximately 13,900 cal yr BP. It is possible that the increase in temperatures contributed to the melting of dead ice, causing an increase in the watering of the basin in the period under consideration.

However, this stage of the basin evolution probably did not last long—several decades—since already from 13,878 ± 150 cal yr BP (depth 180 cm) there is a change in the hydro regime: signs of swamping appear, fixed by the spread of acidophilic algae and the disappearance (dissolution) of diatom valves. The distribution of tree species is decreasing, while the number of juniper and grasses—wormwood and sedges—is increasing, which may reflect a cooling in the temperature trend.

The preservation of such a regime is noted up to the time interval corresponding to 13,829 ± 150 cal yr BP (depth 176 cm), where signs of another change in hydro conditions appear. Since then, there has been a decrease in the acidity of the water, most likely due to an increase in the flow of the reservoir, as evidenced by the appearance of freshwater sponges that prefer not-stagnant water. The biological productivity of the basin is average, but the number and diversity of diatoms is growing. Probably, the depth of the reservoir is increasing, as evidenced by the increase in the number and diversity of epiphytic diatoms. Molluscs are becoming more abundant. Again, due to pine and birch, the proportion of tree species in the vegetation cover is growing. At the same time, the constant presence of heliophytes (*Juniperus* and *Helianthemum*) in the spore-pollen spectrum indicates the openness of the vegetation in the study period. Sedges predominate among herbs, and *Sphagnum* and *Equisetum* appear, which may indicate sufficient soil moisture.

During the time period between 13,753 ± 140 and 13,713 ± 140 cal yr BP (depth 169–165 cm), a new stage is observed, both in the formation of the basin and in the development of adjacent landscapes. Epiphytic eutrophic species of diatoms still dominate, but the bioproductivity of the reservoir is halved. The influx of sandy material into the basin increases, and swamping processes occur. There are noticeable changes in the structure of plant communities, which may indicate a decrease in air temperature while maintaining humidity. The number of pine is sharply reduced and the number of birch is increasing. More Poaceae appear in the herbaceous cover. At the same time, the increased values of pollen and spores of *Thalictrum*, *Typha*, and *Equisetum* testify to the presence of humid habitats.

The next stage in the evolution of the basin and surrounding landscapes lasting from; 13,713 ± 140 to 13,638 ± 140 cal yr BP (depths 165–158 cm) is accompanied by a trend towards a decrease in trophicity and bioproductivity of the reservoir, and is characterized by a decrease in the diversity of microfossils. At the beginning of the stage, peat formation occurs (13,680 ± 130 cal yr BP). The content of green algae is reduced to a minimum. At the same time, the number and diversity of freshwater sponges at the end of the interval is increasing, which testifies to deepening of the basin again. The vegetation cover undergoes some changes—the amount of pine in the forest stand increases, the content of juniper and dwarf birch in the spectrum gradually decreases to a minimum, and Cyperaceae replace Poaceae, again predominate in the composition of herbaceous communities. Ranunculaceae reach a maximum in the first half of the period, but disappear in the second. *Equisetum* disappears from the spectrum, while *Sphagnum* reaches higher values.

At the next stage of basin development, from 13,638 ± 140 to 13,538 ± 160 cal yr BP (depths 158–148 cm), the composition of diatoms somewhat changes: the proportion of oligotrophic species slightly increases and the ratio between eutrophic and mesotrophic species changes in favor of the latter. The appearance of a small number of brackish-water species with great caution suggests the possibility of (short-term?) water exchange with the BIL, and the latter with the world ocean. The number of sponges continues to increase, while ostracods are almost completely disappearing. Some changes of environmental conditions at 13,609 ± 150 cal yr BP cause the temporal disappearance of molluscs, which recover at 13,559 ± 160 cal yr BP again. The dynamism of the formation of the vegetation cover during this period, apparently, reflects the instability of climatic conditions. The contribution of arboreal vegetation is high (up to 75%) but passes a sharp and short-lasting decline (to 55%) at 13,559 ± 160 cal yr BP. *Betula nana* during this period has a maximum value throughout the sequence (3%). *Juniperus* re-appears as well as *Artemisia*, Chenopodiaceae, and Poaceae. Ferns have a peak coinciding with the maximal spreading of pine trees.

A new stage in the evolution of the basin, corresponding to the time period between 13,538 ± 160 and 13,469 ± 170 cal yr BP (depths 148–141 cm), may reflect some major warming. The trophism of the basin again increases. After a slight reduction of tree species in the vegetation cover at about 13,498 ± 160 cal yr BP, their number grows. Probably, during this period, real forests could have been existed, since the amount of tree species reaches 80% in the spore-pollen spectrum. *Sphagnum*, *Equisetum*, and Polypodiaceae testify to the humidity of forests. The number of *Thalictrum* and aquatic plants in general is increasing. Possibly, overgrowth of the banks of the basin is observed, as evidenced by the results of algo-zoological analysis.

Noticeably, the eight stages considered in the formation of the basin and its surrounding landscapes during 14,038–13,469 cal yr BP, being plotted against NGRIP δ^18^O, cover only two events, GI-1d and GI-1c, in the Bølling–Allerød [2]. This clearly demonstrates the high dynamism and complexity of changes in local environmental systems in the Lateglacial in general, and in the first half of the Allerød, in particular, when climatic fluctuations were superimposed on such processes as post-glacial isostatic rebound and formation of hydrographic net. Biotic components of environmental systems can respond to changes of other than temperature factors, such as nutrient status, pH, light conditions, and lake depth, but climatic changes and, in particular, temperature have an over-riding influence [32,33]. The responses of the different groups of organisms to the same climatic event can be asynchronous. Thus, aquatic invertebrates, algae, and zooplankton or insects can react to the change of environmental conditions fast and direct [32,34,35]. Grasses seem to be also a sensitive vegetation dynamics indicator, while arboreal vegetation needs decades to migrate or expand their populations. In the case of the Kulikovo section, we observed some overlap and asynchronicity of the biotic responses as well, though the fact of the reaction of different organisms to that or another factor provided the opportunity to identify the clear stages in the evolution of the studied environmental system. The obtained results also give grounds to assume the existence of repeated short-term (several decades) temperature fluctuations at the end of the Older Dryas and in the first half of the Allerød.

## 5. Conclusions

Data obtained from geochronological, lithological, diatom, algo-zoological, and palynological analyses on the Kulikovo sediment section (south-eastern Baltic Sea coast) provide a reconstruction of local aquatic and terrestrial biocenoses and their response to short-term warmings and coolings that took place between 14,038 ± 140 and 13,469 ± 170 cal yr BP. The research has revealed a complex dynamics of environmental systems during the Older Dryas and the first half of the Allerød. During the GI-1d and GI-1c climatic events, the local biota underwent numerous changes, which resulted in eight stages of the basin evolution, indicated by transformation of the hydrological regime and successions of plant communities from the pioneer swampy vegetation to park and real forests. These changes in aquatic and terrestrial biota most probably were related to the climatic fluctuations that could have had a duration of several decades.

## Figures and Tables

**Table 1:** Geochronology of the Kulikovo section.

Sample	Depth, cm	Material	Age, ^14^C	Model Age, cal yr BP
LuS-18463	45	macroremains (wood)	10,940 ± 60	12,773 ± 240
LuS-18462	106	macroremains (wood)	11,060 ± 60	13,102 ± 160
LuS-18461	163	macroremains (wood)	11,790 ± 60	13,693 ± 130
LuS-18460	186	macroremains (wood)	11,980 ± 80	13,957 ± 140
LuS-17811	192	sediment (gyttja)	12,200 ± 60	14,038 ± 160

## Data Availability

Applicable upon request.
